# Combination of varenicline and nicotine patch for smoking cessation: A case report

**DOI:** 10.1002/ccr3.2332

**Published:** 2019-07-25

**Authors:** Kelly M. Young, James M. Davis

**Affiliations:** ^1^ Duke University Health System DUAP Oxford Family Practice Oxford North Carolina; ^2^ Duke Cancer Institute Duke University School of Medicine Durham North Carolina

**Keywords:** chantix, nicotine, nicotine dependence, nicotine replacement therapy, smoking, smoking cessation, tobacco, varenicline

## Abstract

Standard pharmacotherapy approaches to treat tobacco use may be ineffective in people with high nicotine dependence. Individuals with high nicotine dependence may be good candidates for a new treatment approach—combination of varenicline and nicotine patch.

## 1 BACKGROUND

Smoking is the number one cause of preventable disease and death in the United States.^1^ There are now seven FDA‐approved medications that are effective in helping individuals quit smoking,^2^ including varenicline, found in a large meta‐analysis to be the most effective monotherapy available,^3^ and nicotine replacement, the most commonly used medication for smoking cessation.^2^ These treatments, however, are often ineffective,^3^ and new, more effective medication regimens are needed.

There is currently considerable interest in the potential efficacy of the combination of varenicline and nicotine patch for smoking cessation. This is not an intuitive combination, because the nicotine patch acts as a full agonist at the nicotinic‐acetylcholine receptor,^4^ whereas varenicline acts as a long‐acting partial agonist at the nicotinic‐acetylcholine receptor,^3^, ^4^ As such, there is concern that varenicline might block nicotine from binding to nicotinic‐acetylcholine receptors, rendering the nicotine patch ineffective.^2^, ^4^ In practice, however, there is growing evidence that the nicotine patch may add to the therapeutic effect of varenicline.^5^ This may occur because varenicline only occupies a portion of available nicotinic receptors, thereby allowing nicotine from the nicotine patch to bind to the remaining free receptors, producing a therapeutic effect.^5^, ^6^ We present the case of a 71‐year‐old patient with a history of heavy smoking who successfully achieved smoking abstinence with a combination of high‐dose varenicline and nicotine patch.

## 2 CASE PRESENTATION

Our patient was a 71‐year‐old male with a medical history of lung cancer, chronic obstructive pulmonary disease (COPD), and bipolar disorder. He reported smoking 40 cigarettes per day for 56 years (112 pack‐year smoking history). He had attempted to quit smoking more than 15× over the course of his life and stated that he had used varenicline, nicotine patch, and nicotine gum in the past, but that none had led to significant suppression of smoking urges or sustained smoking abstinence.

The patient was seen in a university setting within a specialized tobacco treatment clinic and scored a 10 (the highest possible score) on the Fagerstrom Test of Nicotine Dependence, indicating high nicotine dependence. Due to his history of minimal response to standard‐dose monotherapies, the patient was started on high‐dose, combination nicotine replacement (two 21 mg patches plus 4 mg nicotine lozenges).

Three weeks later, the patient returned for a follow‐up visit; he reported adherence to his combination nicotine replacement but no significant reduction in cigarettes per day or smoking urges. The patient was then transitioned to standard varenicline dosing (1 mg twice per day), plus 4 mg nicotine lozenges. Two weeks later, the patient returned for third visit and was found to be compliant with this new medication regimen; he stated that he tolerated it well but had no reduction in smoking or smoking urges. The patient wanted to make a quit attempt and was given a quit day in 3 weeks.

The patient returned for a fourth visit on his quit day. He had not quit and had no reduction in urges. He was then prescribed varenicline (standard dose) plus 21 mg nicotine patch. Three weeks later, he returned for a fifth visit and reported that smoking urges had significantly improved on a combination of varenicline and nicotine patch and that he had quit smoking. Carbon monoxide (CO) breath testing was consistent with smoking abstinence (CO = 1 ppm). At a 12‐week postquit visit, he reported sustained total abstinence, confirmed by CO testing, and no medication side effects.

## 3 DISCUSSION AND CONCLUSIONS

Several randomized clinical trials have assessed the combination of varenicline and nicotine patch vs. varenicline alone and showed mixed results. In 2013, Hajek et al randomized 117 individuals who smoked and reported that the combination of varenicline and nicotine patch was well tolerated but was not more effective than varenicline alone for smoking cessation. Participants attended standard weekly support sessions following withdrawal‐oriented treatment protocol as provided by the NHS Stop‐Smoking Service.^3^ In 2014, Ramon et al randomized 341 individuals who smoked and showed that the combination was well tolerated, but again with no differences between groups. A post hoc analysis, however, showed that for those who smoked 30 or more cigarettes per day, the combination treatment was superior to varenicline alone (OR 1.46; 95% CI 1.2‐2.8). At each visit, smokers participated in behavioral counseling sessions that had been previously standardized. Sessions lasted 10 to 15 minutes each and were based on motivational interviewing.^6^ In 2014, Koegelenberg et al randomized 435 individuals who smoked and found that smoking cessation was superior with varenicline and patch vs. varenicline with continuous abstinence of 49.0% vs 32.6%; OR, 1.98; 95% CI, 1.25‐3.14; *P * = 0.004. Ten minutes of smoking cessation counseling, based on the 2008 update of the US Public Health Service guidelines, was provided to all participants at each visit.^4^ Unlike the previous two studies, Koegelenberg started nicotine patch treatment 2 weeks prior to the quit day. In 2015, Chang et al conducted a meta‐analysis of these three studies showing that across all three trials, combination of varenicline and nicotine patch was superior to varenicline alone, though the difference was small when the effects of using prequit patch was removed.^5^

An important question that arises from this data is whether there may be a subpopulation that responds robustly to the combination of varenicline and nicotine patch. There are now two studies that show that combined varenicline and bupropion is superior to varenicline alone,^7^, ^8^ but the effect appears to be driven primarily by a benefit of combination treatment in smokers with high nicotine dependence and shows little or no effect in individuals with low or moderate nicotine dependence.^9^

This case adds to the existing literature because it provides an example of a heavy smoker with high nicotine dependence who did not respond to nicotine patch or varenicline alone but did respond to these medications when used in combination. The case suggests that mixed results in the literature may be due to the use of a general population and that a subpopulation, for example those who are male, heavy smokers, or have high nicotine dependence, may in fact respond quite favorably to this combination treatment. Other factors to consider may include the presence of physical or psychiatric co‐morbidities (ie, history of lung cancer, COPD, bipolar disorder).

This patient, who smoked heavily and had high‐level nicotine dependence, represents an extreme point along a continuum of those who smoke. As an extreme case with a nonambiguous response, this patient may provide an example of a subpopulation who responds well to this combination therapy. As a case study, there is no control group; the case does, however, provide a natural “within subject comparison,” in that the patient was at first unable to quit smoking using varenicline and nicotine patch individually and then later was successful in quitting using a combination of varenicline and nicotine patch. Another relevant component of the case is that the patient experienced a reduction in urges and withdrawal symptoms on the combination of varenicline‐nicotine patch, which he did not experience while using these medications individually. This case report is consistent with the findings of Ramon et al, who found significantly higher abstinence rates with varenicline plus patch vs. varenicline alone, but only in individuals who smoked ≥30 cigarettes per day^6^; our patient smoked 40 cigarettes per day. Given the mixed findings from prior studies on the efficacy of varenicline plus nicotine patch, this study suggests the possibility that participants with specific characteristics, for example, those who smoke more heavily or those with high nicotine dependence, may be appropriate candidates for the combined use of varenicline and nicotine patch and that future study may benefit from additional focus on these populations.

## CONFLICT OF INTEREST

The authors have no conflicts of interest to report.

## AUTHOR CONTRIBUTIONS

Kelly M. Young and James M. Davis were fully involved with development, writing, and review of the case report.

## CONSENT FOR PUBLICATION

We obtained consent from the patient and it is on file at the Center for Tobacco Cessation.
